# Editorial: Male hypogonadism: need for reclassification?

**DOI:** 10.3389/fendo.2024.1514350

**Published:** 2024-11-11

**Authors:** Rossella Cannarella, Richard Quinton, Aldo E. Calogero

**Affiliations:** ^1^ Department of Clinical and Experimental Medicine, University of Catania, Catania, Italy; ^2^ Glickman Urological & Kidney Institute, Cleveland Clinic Foundation, Cleveland, OH, United States; ^3^ Department of Metabolism, Digestion & Reproduction, Imperial College London, London, United Kingdom; ^4^ Northern Region Gender Dysphoria Service, Cumbria, Northumberland, Tyne & Wear NHS Foundation Trust, Newcastle, United Kingdom

**Keywords:** hypogonadism, hypogonadotropic hypogonadism (HHG), reversal, classification, central hypogonadism

First systematically described in modern times by Albright in 1941 as a clinical syndrome caused by gonadal dysfunction (1), the concept of male “eunuchoidism” dates back to biblical times and beyond (2). Although the diagnosis has been usually expressed in terms of testosterone deficiency, it is now increasingly recognized that subfertility due to impaired spermatogenesis represents another key feature (3; Munari et al.). Numerous causes of hypogonadism have been described, albeit broadly classified into forms with hypothalamic-pituitary (hypogonadotropic, central, or secondary), and gonadal (or primary) etiologies; in turn divided into congenital and acquired forms.

Over recent years, the so-called functional forms of hypogonadism have been recognized, including age- and obesity-related causes, albeit some investigators prefer the concept of non-gonadal illness (4). The Massachusetts Male Aging Study identified a decline in serum testosterone levels with advancing age and accentuated by the presence of comorbidities (5). It has been estimated that obesity and diabetes mellitus are found in approximately one-third of men with sexual symptoms, hypotestosteronaemia and inappropriately normal gonadotropin values ​​ (6). However, although the European male Aging Study found this biochemical signature in 11.8% of older men, only 2.2% had the associated triplet of sexual symptoms (reduced libido, loss of spontaneous erections and impaired quality of erections) that conferred a clinical diagnosis (7). Moreover, the decline in luteinizing hormone (LH)-mediated serum testosterone concentrations was overwhelmingly determined by accumulating comorbidities (especially obesity), with no direct impact of chronological aging *per se*.

Added to this has been the rapid advance in identifying ever more genetic associations of central hypogonadism using next-generation sequencing (NGS), although many genes have yet to be tested in animal models. The complexity of these results is also intriguing, with many mutations being neither completely “white” (non-pathogenic) nor “black” (pathogenic), and the data suggests that may include various shades of “gray”. NGS, for example, allows detection of mutations or polymorphisms that could confer predisposition to developing central hypogonadism in the presence of specific environmental triggers, such as excessive weight loss (8), critical illness, exposure to stressors, obesity, diabetes mellitus, etc, i.e. blurring the distinction between functional and organic hypogonadism.

Thus, hypogonadism may not always be a condition of simple definition and identification, and new classifications may be necessary to fully understand its various manifestations and forms. In this Research Topic, we have assembled articles that describe less conventional forms of hypogonadism in both males and females.

A literature review by Barbagallo et al. signposts the role of genetics in determining individual susceptibility to the onset of acquired functional hypogonadism in females, noting the numerous genetic mutations identified patients who presented following drastic weight loss. These observations likewise indicate a strong interplay between genetic factors and the environment.

Fertile eunuch or Pasqualini syndrome was first described in 1950 and is characterized by congenital hypogonadotropic hypogonadism with partial onset of spontaneous puberty (testicular volume >8 ml), normal follicle-stimulating hormone (FSH), and testicular histology consistent with normal spermatogenesis (9). The article by Dwyer et al., represents the largest study of fertile eunuch syndrome published to date.

They examined the frequency of reversal (10; 13) among a cohort of patients with congenital central hypogonadism studied at the Reproductive Endocrine Unit of the Massachusetts General Hospital, Boston, MA, USA (1980-2020); dividing the cohort into males lacking puberty at presentation (n = 139), with partial puberty (n = 63), and those with fertile eunuch syndrome (n = 36). The classification highlights how central hypogonadism can manifest itself completely or partially, depending on whether GnRH pulsatility is absent or merely impaired; in particular, low frequency pulses favor FSH secretion, which may underpin the preserved spermatogenesis observed in Pasqualini syndrome.

Moreover, Dwyer et al. observed that, whilst the overall prevalence of reversal is 10‒15% among males with congenital central hypogonadism (11), this varies according to the original subtype at presentation, from 5% of those lacking puberty, through 13% in those with partial puberty to 44% in fertile eunuch syndrome. The classic definition of hypogonadism does not include forms with partial puberty or with preserved spermatogenesis, which are instead compatible with the presence of only partially compromised genetics (for example polymorphisms, compound heterozygosities, etc.). This study likewise highlights the need for a reclassification of hypogonadism.

The article by Aung et al. highlights how difficult it is to differentiate during adolescence between forms of partial central hypogonadism lacking a non-reproductive phenotype (such as anosmia, clefting, deafness, etc) and self-limiting delayed puberty. The authors suggest that the clinical-biochemical combination of micropenis and low levels of inhibin B can be helpful to the differential diagnosis.

This Research Topic also includes an article that fits into the ongoing debate regarding medical *versus* surgical treatment of cryptorchidism. Through a retrospective design, Sun et al. observed the same efficacy results (in terms of testicular volume and spermatogenesis efficiency) in ex-cryptorchid patients treated with medical therapy or with combined medical and surgical therapy. They conclude that medical therapy is a safer, less invasive, and less expensive option than surgical therapy. The medical therapy that patients underwent in this study included both human chorionic gonadotropin (hCG)-monotherapy and pulsatile Gonadotropin-releasing hormone (GnRH) (equivalent to combined hCG+FSH therapy). It remains to be seen whether one modality is associated with better outcomes than the other. What is the best therapeutic approach when it comes to medical therapy of cryptorchidism remains a hot topic today (12).

We hope that the articles in this Research Topic will be useful to readers in understanding how heterogeneous what we define as hypogonadism is and how much progress still needs to be made to fill the existing gaps. We truly hope that some of these articles will help us understand, at least in part, some of the open questions and inspire *ad hoc* studies that further improve our knowledge on this Research Topic and that this will lead to a better and more comprehensive reclassification of “central hypogonadism”.
